# Suppression of Hedgehog signaling is required for cementum apposition

**DOI:** 10.1038/s41598-020-64188-w

**Published:** 2020-04-29

**Authors:** Hwajung Choi, Yudong Liu, Liu Yang, Eui-Sic Cho

**Affiliations:** 1 Cluster for Craniofacial Development and Regeneration Research, Institute of Oral Biosciences, Chonbuk National University School of Dentistry, Jeonju, 54896 South Korea; 2 Department of Histology and Embryology, Bengbu Medical College, Bengbu, Anhui P.R. China

**Keywords:** Bone development, Musculoskeletal development

## Abstract

Hedgehog (Hh) signaling plays a broad role in the development of many organs including bone and teeth. It is noted that sustained Hh activity in osteoblasts negatively regulates postnatal development in mice. However, it remains unknown whether Hh signaling contributes to cementum formation. In this study, to define the roles of Hh signaling in cementum formation, we analyzed two kinds of transgenic mouse models for Hh signaling activation designed by the inactivation of Suppressor of Fused (*Sufu*), a negative regulator of Hh signaling, (*Sufu*^*OC*^) and a forced endogenous activation of *Smo* (*SmoM2*^*OC*^) under the control of *osteocalcin* (*OC*) promoter-driven Cre recombinase. Interestingly, cellular cementum apposition was remarkably reduced in both mutants. Consistently, matrix formation and mineralization ability were down-regulated in OCCM-30, a cementoblast cell line, following treatment with a pharmaceutical Smo agonist. In addition, reductions in Osx expression and β-catenin activity, which are critical for cellular cementum formation, were also detected *in vitro*. Furthermore, the compound mutant mice designed for the stabilization of β-catenin with both Hh-Smo signaling activation in cementoblasts revealed a complete restoration of defective cellular cementum. In addition, Wnt antagonists such as Sostdc1 and Dkk1 were also induced by Smo activation and played a role in the reduction of Osx expression and β-catenin activity. Collectively, our data demonstrated that Hh signaling negatively regulates cementum apposition in a Wnt/β-catenin/Osx-dependent manner.

## Introduction

Cementum covers the tooth root and allows for the attachment of periodontal ligaments between the roots and alveolar bone and plays adaptive roles in supporting the tooth in its proper occlusal position^1^. Developmentally, cementum is formed as a heterogeneous connective tissue that differs with respect to location, structure, function, rate of formation, chemical composition, and degree of mineralization^2^. Acellular extrinsic fiber cementum is mainly found on the cervical and middle portions of the root. Cellular intrinsic fiber cementum mainly located apically is a unique avascular hard tissue that does not undergo continuous remodeling but continuously grow in thickness throughout life^1^. It has been known that cementum has the dynamic and highly responsive features critical for maintaining occlusal relationships, the integrity of the root surface, and its function in tooth support^1^.

Recently, there has been increasing evidence to support key interactions between Osterix (Osx), β-catenin, and pyrophosphate (PPi) in cementum development and homeostasis. Osx has been reported as an important factor orchestrating overall tooth root formation. Osx regulates odontoblast differentiation, maturation, and root elongation including cementum formation^3–6^. Excessive cementum formation occurs in transgenic mice where β-catenin is constitutively stabilized^7^. β-catenin induces cementoblast differentiation and boosts matrix secretion through a reciprocal interaction with Osx during cellular cementum formation^6^. It is suggested that a local balance between Osx and PPi is related to the determination of the cementum type in which local PPi suppresses matrix accumulation and further mineralization. We have been reported an antagonistic interaction of PPi with Osx and that their relationship is under the synergistic influence of FGF signaling during cementum formation^8^.

The roles of Hedgehog (Hh) signaling pathway has been known to regulate the growth and morphogenesis in the development of several organs^9,10^. The Hh ligand binds to an Hh receptor, patched (Ptc), stopping the suppression of the transmembrane protein, smoothened (Smo), which stabilizes the glioma-associated oncogene (Gli) transcription factor. Interestingly, Hh signaling targets Ptc1 and Gli1 in vertebrates, and the presence of Ptc1 and Gli1 transcripts indicates the functional activation of Hh signaling^11,12^. It is well-known that Suppressor of Fused (Sufu) negatively regulates Hh signaling by direct binding to the Gli protein^13^. When the cells lack Sufu, the Hh signaling pathway is maximally activated in a ligand-independent manner^14^. However, the regulation system for Hh signaling through Sufu seems having more complicated connection with other signaling. Sufu may act as a dual regulator of Hh and Wnt/β-catenin signals and integrate the multiple pathways during development^15^. It has been previously reported that Sufu plays a role on the repressive regulation of Hedgehog activity in preosteogenic mesenchyme to ensure osteogenesis in calvarial bone formation^16^. We have reported that sustained Hh activity in osteoblasts inhibits postnatal bone development by suppressing the gene expression of bone formation regulatory factors in mice^17^.

The aim of this study was to investigate the developmental regulation of Hh-Smo signaling in cementum formation during tooth development. We investigated the role of Hh-Smo signaling in cementum formation using multiple approaches and demonstrated that the activation of Hh-Smo signaling in cementoblasts leads to defective cementum formation through the inactivation of Osx and β-catenin.

## Results

### Hh-Smo signaling activation in cementoblasts leads to a reduction in cellular cementum

**Figure 1:**
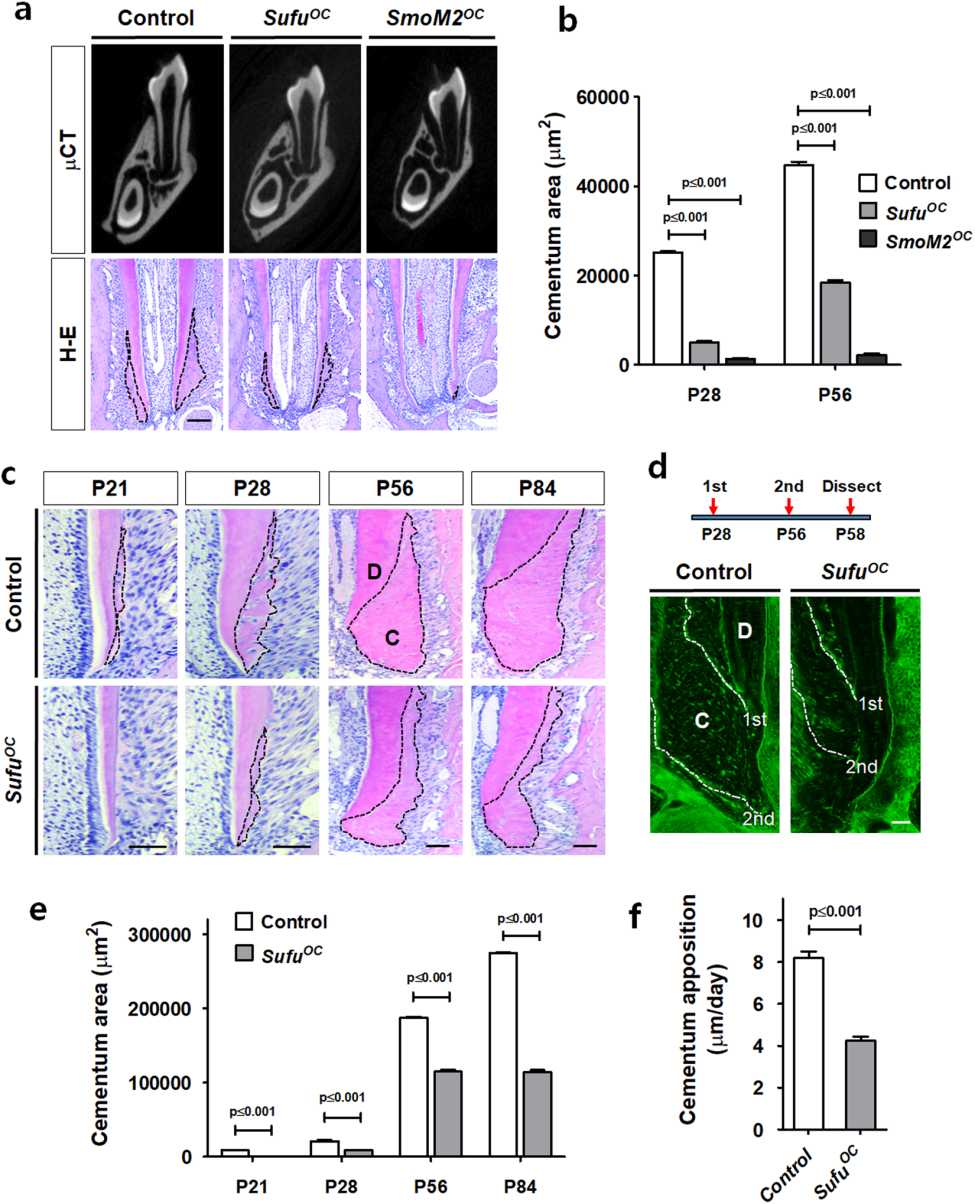
Hh-Smo signaling activation in cementoblasts leads to a reduction in cellular cementum. (**a**) Morphological changes in the tooth root and the apical cellular cementum (indicated by dotted lines) of *Sufu*^*OC*^, *SmoM2*^*OC*^ mutant, and the control mice were compared by μCT and H-E staining at P28 of age. Scale bar; 100 μm (H-E). **(b)** The cementum area was analyzed with the distal root of the mandibular first molar at P28 and P56. **(c)** Chronological changes in the cellular cementum volume (indicated by dotted lines) of *Sufu*^*OC*^mutant and the control mice were compared with H-E-stained tissue sections of the distal root of the first molar at the indicated age. P21, postnatal day 21; D, dentin; C, cementum. Scale bar; 100 μm. **(d)** The apical cellular cementum apposition rate was compared with the distal root of the mandibular first molar at P58 after double-fluorochrome labelling. The date of injection and the fluorochrome line (indicated by white dotted lines) were indicated. D, dentin; C, cementum. Scale bar; 50 μm. **(e)** The cementum area was analyzed with H-E-stained tissue sections in Fig. 1c. **(f)** The cementum apposition rate was quantified with the distal root of the mandibular first molar after double-fluorochrome labelling. Significance was assigned for *p*-values as indicated in the graph.

To study the gene regulatory networks downstream of the Hh-Smo signaling cascade in cementum formation during tooth development, we generated two kinds of mice for Hh-Smo activation, which are conditionally inactive for *Sufu* (*Sufu*^*OC*^) and constitutively active for *Smo* (*SmoM2*^*OC*^) in *OC*-positive dental mesenchyme. We confirmed the Hh gain-of-function in the dental tissue of *Sufu*^*OC*^ mice through Sufu and Ptc1, a downstream target of Hh signaling, expression by immunohistochemical (IHC) staining (Supplementary Fig. S1). Root length and dentin thickness were generally decreased by both Smo-activated models. The changes were more remarkable in *SmoM2*^*OC*^ than *Sufu*^*OC*^ mice as shown by microcomputed tomography at postnatal 28 days (P28) (Fig. 1a) and H-E staining at P14 (Supplementary Fig. S2a). There is no change in tooth eruption between both models. Most interestingly, both Hh-Smo signaling activation mice displayed a reduction in cellular cementum mass with a shorter root length compared to the control, as observed by μCT and H-E staining at P28 of age (Fig. 1a). More dramatic changes in the gross cellular cementum mass of the mandibular first molar were observed in *SmoM2*^*OC*^ than in *Sufu*^*OC*^ mice and the gap in the amount of cementum mass between the two mutants increased with aging as analyzed by the cementum area up to P56 (Fig. 1b and Supplementary Fig. S2b). Furthermore, the reduction of cellular cementum mass in *Sufu*^*OC*^ mice was not fully recovered during further development, as observed by H-E staining of the dental tissue up to P84 (Fig. 1c,e). To address whether Hh-Smo signaling activation plays a role in controlling the matrix apposition rate in cementogenesis, a fluorochrome labeling assay was used. The distance between the double-fluorochrome labeling lines, reflecting the rate of cellular cementum formation, was much shorter in *Sufu*^*OC*^ mice (4.2 μm/day) than in the control mice (8.2 μm/day) (Fig. 1d,f). To clarify the relationship between Hh-Smo signaling and cellular cementum formation, we have also analyzed Smo inactivation mice, which are conditionally inactive for *Smo*(*Smo*^*OC*^) controlled under the same *OC*-Cre recombinase. Different with our expectation, *Smo*^*OC*^ mutant mice exhibited normal development of cellular cementum whereas *SmoM2*^*OC*^ mutant mice exhibit clear reduction in cellular cementum apposition (Supplementary Fig. S2c). The results indicate that inactivation of endogenous Smo is not enough to promote cementum apposition. Taken together, our results strongly suggest that Hh-Smo signaling is repressed for the proper formation of cellular cementum at the apex of the tooth root.

### Reduced matrix formation and mineralization rates of cellular cementum by Hh-Smo signaling activation in cementoblasts

**Figure 2:**
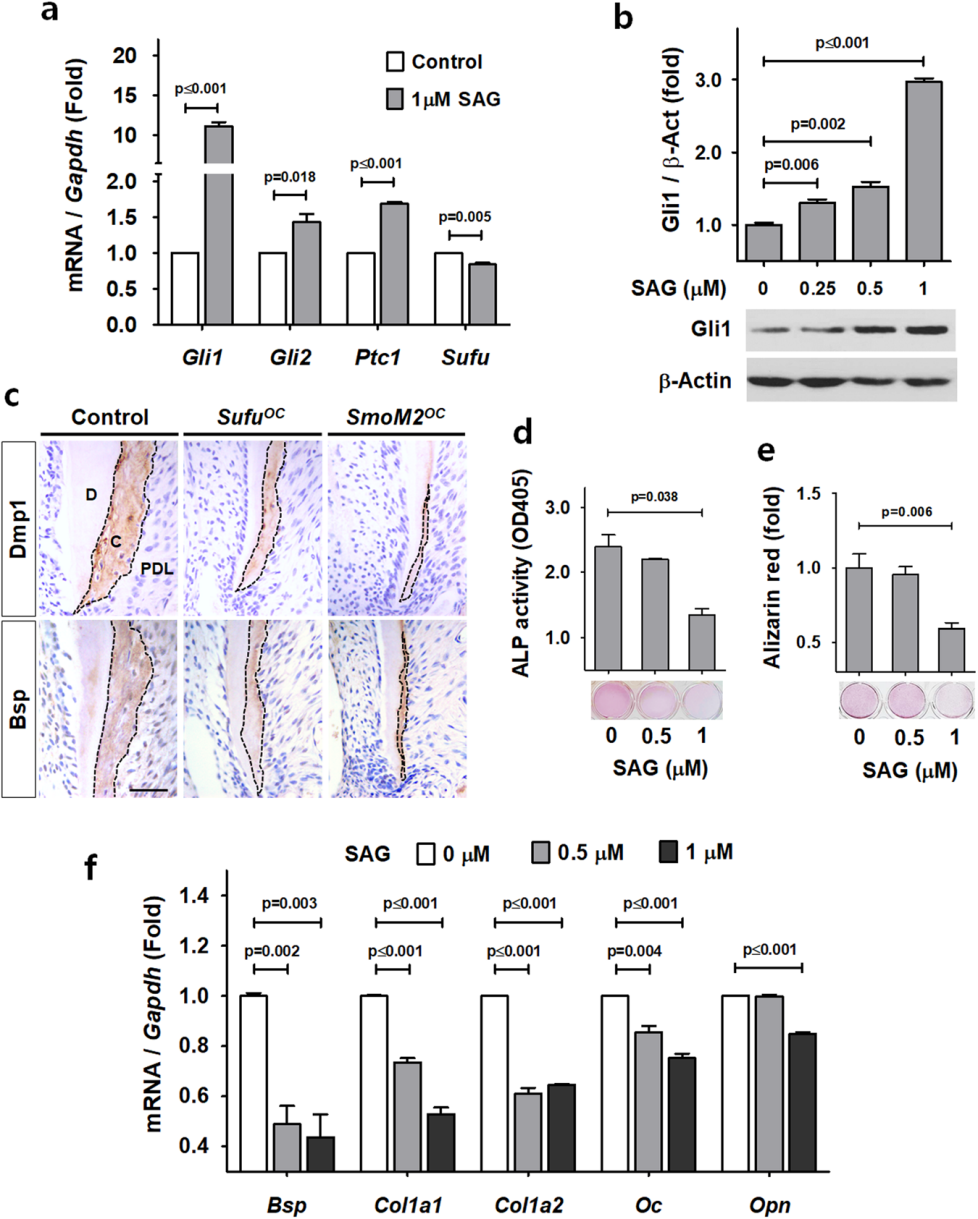
Reduced matrix formation and mineralization rates of cellular cementum by Hh-Smo signaling activation in cementoblasts. (**a**) The mRNA transcript levels were analyzed by real-time qPCR. RNA was isolated from OCCM-30 cells treated with 1μM SAG for 72 hours. **(b)** The protein levels of OCCM-30 cells treated with the indicated concentrations of SAG for 24 hours were analyzed by Western blotting. Samples shown in Western blotting are from the same experiment, and the gels/blots were processed under the same experimental conditions. β-Actin was used as a loading control. Cropped images are displayed here; the original full-size blots are presented in Supplementary Fig. S6a. **(c)** Molecular changes in the apical cementum (indicated by dotted lines) of *Sufu*^*OC*^ and *SmoM2*^*OC*^ mutant mice and the control mice were detected by IHC staining with the distal root of the mandibular first molar at P28. C, cementum; D, dentin; PDL, periodontal ligament. Scale bars: 100 μm. **(d**,**e)** Alkaline phosphatase (ALP) activity **(d)** and mineralization ability by Alizarin red S staining **(e)** were analyzed with OCCM-30 cells treated with OM and the indicated concentrations of SAG for 4 days. **(f)** The mRNA transcript levels were analyzed by real-time qPCR. RNA was isolated from OCCM-30 cells treated with the indicated concentrations of SAG for 72 hours. Significance was assigned for *p*-values as indicated in the graph.

To investigate the molecular mechanism of altered Hh-Smo signaling in cementogenesis, we induced Smo activation in OCCM-30 using SAG, a pharmaceutical Smo agonist. The transcript levels of Hh signaling readout genes including *Gli1*, *Gli2*, and *Ptc1*, were increased by the treatment of 1 μM SAG while the transcription of *Sufu* was mildly reduced (Fig. 2a). With the treatment of SAG, Gli1 protein expression was also induced in a concentration-dependent manner (Fig. 2b). Dramatic reductions in the total sum of Bsp and Dmp1, molecular markers of cementum, expression were detected at diminished cellular cementum mass with Hh-Smo signaling activation in cementoblasts by IHC staining of the dental tissue while higher expression in the developing cementum of control mice was detected (Fig. 2c). The activation of Smo via SAG treatment significantly diminished the ALP activity and mineralization rate of OCCM-30 cells in a concentration-dependent manner (Fig. 2d,e). We next determined whether Smo activation in cementoblasts altered the levels of extracellular matrix proteins important for the regulation of cellular cementum. As expected, the transcript levels of matrix proteins including *Bsp*, *collagen 1a1* (*Col1a1*), *collagen 1a2* (*Col1a2*), *OC*, and *Opn*, were reduced by the treatment of SAG in a concentration-dependent manner (Fig. 2f).

### Hh-Smo signaling activation results in reduced Osx expression and β-catenin activity in cementoblasts

We determined whether the cellular cementum deformities of *SmoM2*^*OC*^ and *Sufu*^*OC*^ mice occurred through a resorption process via osteoclasts. Tartrate-resistant acid phosphatase (TRAP) staining of the mandibular first molar from control, *SmoM2*^*OC*^, and *Sufu*^*OC*^ mutant mice revealed that most of the TRAP-positive (TRAP +) osteoclasts were detected at the marginal area of alveolar bone in all three types of mice, while TRAP + osteoclasts around the apical cellular cementum region were barely detected (Supplementary Fig. S3a). TRAP + tissue area and TRAP + osteoclast cell numbers were analyzed where the apical cementum was located as well as the cementum-faced side of the alveolar bone (Supplementary Fig. S3b,c). The amount of TRAP + tissue area and TRAP + osteoclast cell numbers in the apical cementum area did not exhibit a significant difference between control and both Hh-Smo activation mice. These results suggest that the apical cementum phenotype of Hh-Smo signaling activation mice occurred through reduction in newly formed cellular cementum mass and not by a postnatal resorption process.

**Figure 3:**
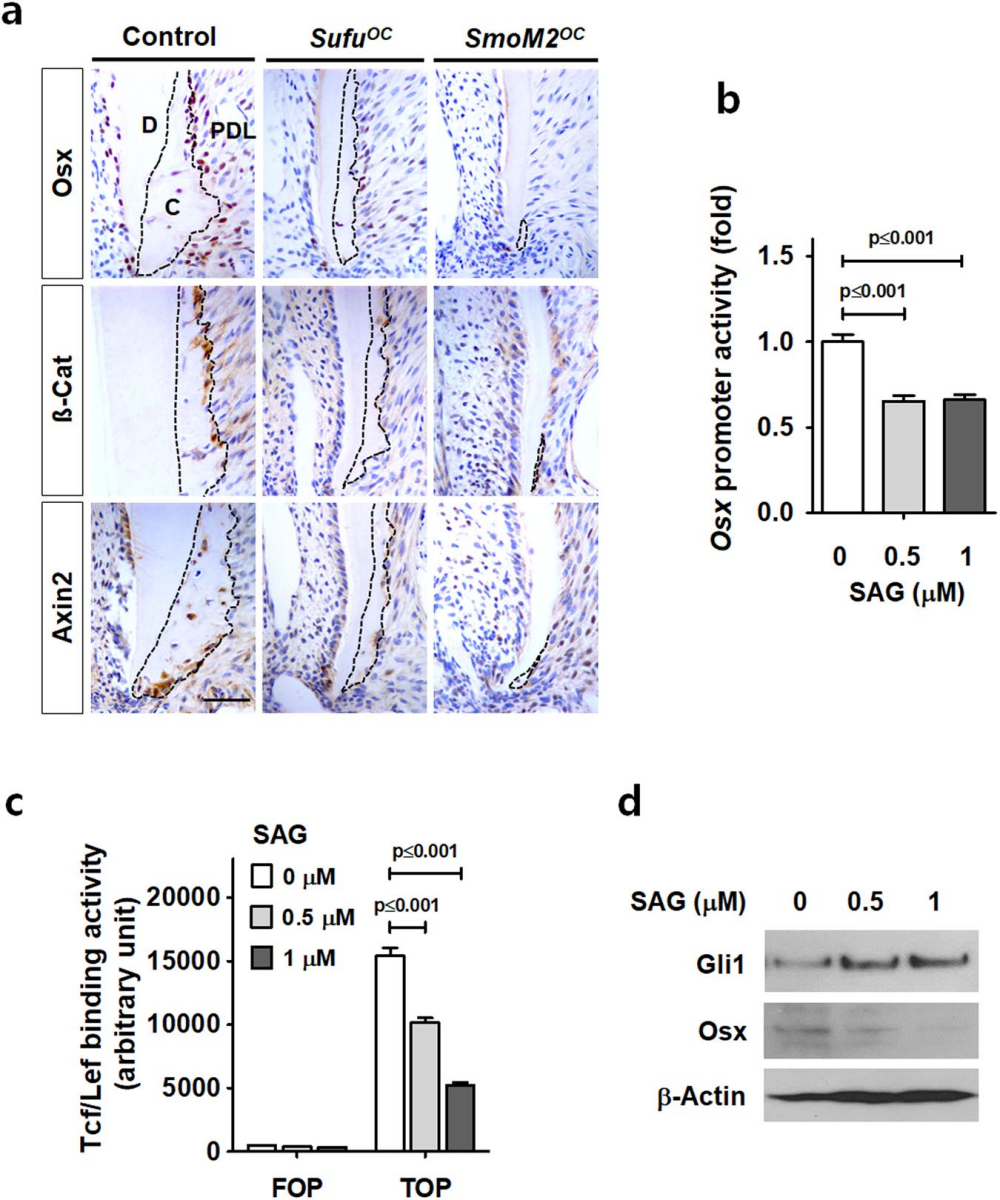
Hh-Smo signaling activation results in reduced Osx expression and β-catenin activity in cementoblasts. **(a)** Molecular changes of Osx, β-catenin and Axin2 in the apical cementum (indicated by dotted lines) were detected by IHC staining with the distal root of the mandibular first molar at P28. C, cementum; D, dentin; PDL, periodontal ligament. Scale bars: 100 μm. **(b and c)** After treatment with gradually increasing concentrations of SAG for 24 hours, luciferase activity driven by the *Osx* (-1269/+91) promoter **(b)** and Tcf/Lef binding activities of β-catenin **(c)** was analyzed using OCCM-30 cells. Significance was assigned for *p*-values as indicated. **(d)** The protein levels of OCCM-30 cells treated with indicated concentrations of SAG in OM for 48 hours were analyzed by Western blotting. Samples shown in Western blotting are from the same experiment, and the gels/blots were processed under the same experimental conditions. β-Actin was used as a loading control. Cropped images aredisplayed here; the original full-size blots are presented in Supplementary Fig. S6b.

Since Osx and β-catenin have been reported to be key regulators in cellular cementum formation^5,7^, we hypothesized that the activities of Osx and β-catenin were reduced by Hh-Smo signaling activation to drive defect cellular cementum formation in *SmoM2*^*OC*^ and *Sufu*^*OC*^ mutant mice. IHC staining showed that Osx, β-catenin, and Axin2, a downstream target of Wnt/β-catenin, were highly expressed in the cementocytes of developing cementum in the control mice at P28 (Fig. 3a). However, dramatic reductions of protein expression in the cementocytes and in the cementum mass were observed in *SmoM2*^*OC*^ and *Sufu*^*OC*^ mutant mice (Fig. 3a). These results imply that the volume of the cellular cementum mass of both mutant mice was regulated in a Wnt/β-catenin/Osx-dependent manner. To confirm the relationship between Osx expression and Smo activation in cementum formation, we analyzed the *Osx* promoter activity and Tcf/Lef binding activity of β-catenin after SAG treatment with OCCM-30 cells. As shown in Fig. 3b,c, Osx expression and β-catenin binding activity were also significantly decreased by SAG treatment *in vitro*. In addition, Smo activation by SAG treatment to OCCM-30 cells reduced the protein level of Osx in a concentration-dependent manner, while the protein level of Gli1 increased concomitantly (Fig. 3d).

### Rescue of impaired cementum through the activation of β-catenin and Osx

**Figure 4:**
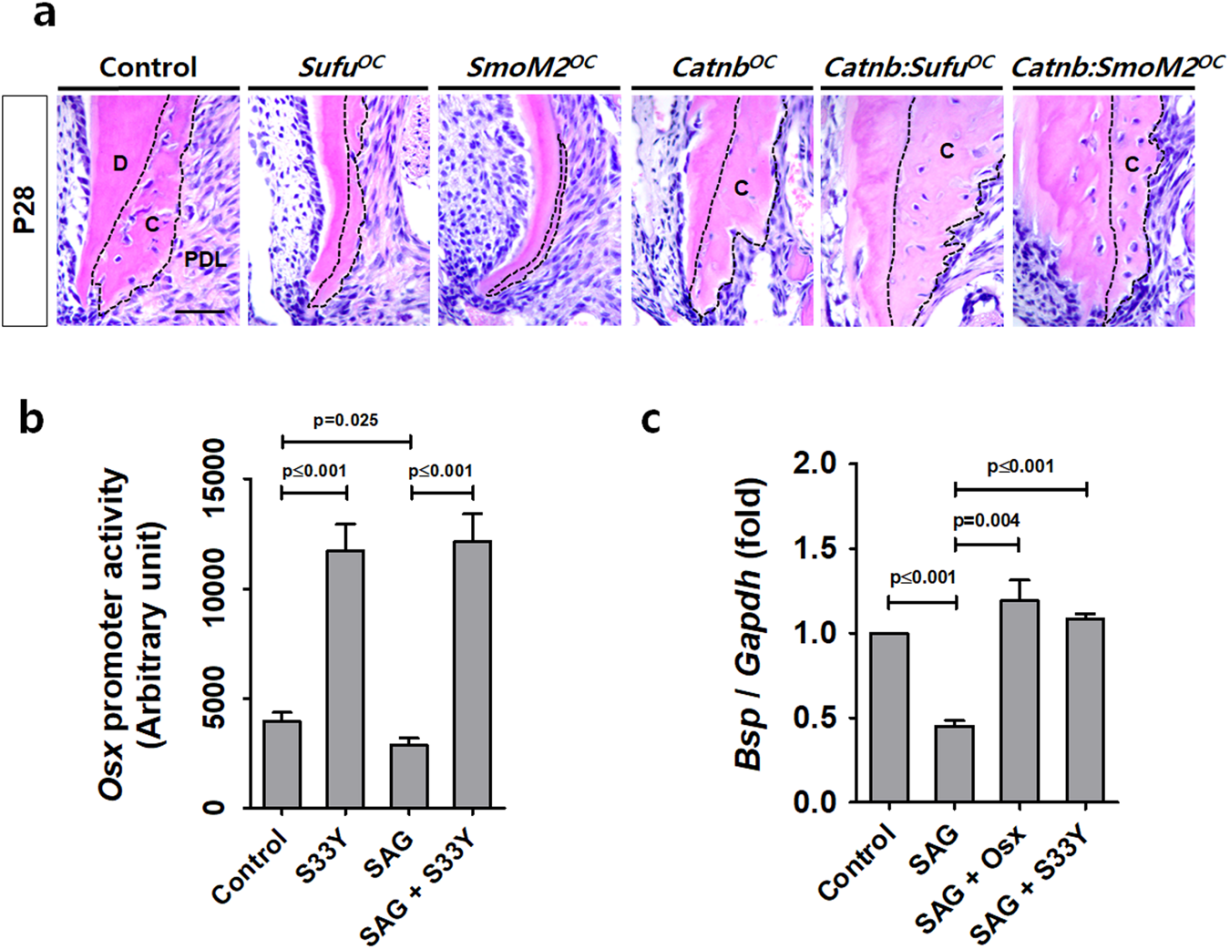
The activation of β-catenin and Osx restores the impaired cementum as a result of Hh signaling activation. (**a**) Histologic changes in the apical cementum (indicated by dotted lines) were detected by H-E staining with the distal root of the mandibular first molar at P28. C, cementum; D, dentin; PDL, periodontal ligament. Scale bars: 100 μm. **(b)** *Osx* promoter activities were analyzed using OCCM-30 cells treated with 1 μM SAG for 24 hours after transfection of mouse *β-catenin* S33Y constructs. **(c)** The *Bsp* transcript levels were analyzed by real-time qPCR. RNA was isolated from OCCM-30 cells treated with 1 μM SAG for 72 hours after transfection of the mouse *Osx* and *β-catenin* S33Y constructs. Significance was assigned for *p*-values as indicated.

We previously reported excessive cementum formation by the stabilization of β-catenin in *OC-Cre:Catnb*^*lox/+*^ (*Catnb*^*OC*^) mice, which have molars with a thicker cellular cementum layer on the root surface including the cervical region^7^. To test whether constitutively active β-catenin in cementoblasts could restore cellular cementum defects from elevated Hh-Smo signaling *in vivo*, we generated and analyzed *Catnb:Sufu*^*OC*^ and *Catnb:SmoM2*^*OC*^ mice, which corresponded to forced β-catenin signaling with Hh-Smo signaling activation in cementoblasts. Interestingly, the apical cellular cementum of *Catnb:Sufu*^*OC*^ and *Catnb:SmoM2*^*OC*^ mice at P28 were dramatically restored by constitutively active β-catenin when compared to the corresponding single mutants (Fig. 4a). To determine whether the forced expression of stabilized β-catenin could induce Osx expression even with Smo activation *in vitro*, we treated SAG to OCCM-30 cells after transfection with active forms of mouse *β-catenin*, a mutated *β-catenin* at Ser-33 *(β-catenin* S33Y)^18^ and analyzed *Osx* promoter activity. As shown in Fig. 4b, the results of the luciferase reporter assay indicate an elevation in *Osx* promoter activity by *β-catenin* S33Y and a reduction in *Osx* promoter activity by SAG treatment. However, the SAG treatment did not inhibit *Osx* promoter activity in the cells with higher β-catenin activity by *β-catenin* S33Y transfection. In addition, we treated SAG to OCCM-30 cells after transfection with mouse *Osx* and *β-catenin* S33Y. Forced β-catenin activity and Osx expression in OCCM-30 cells restored the mRNA transcript level of *Bsp*, a marker of cementum, to the level of control when compared with SAG-treated cells (Fig. 4c). Taken together, the results suggest that the Wnt/β-catenin/Osx signaling pathway inhibits Hh-Smo signaling in cementoblasts during cellular cementum apposition.

### Sostdc1 activated by Hh-Smo signaling inhibits Osx expression in cementoblasts

**Figure 5:**
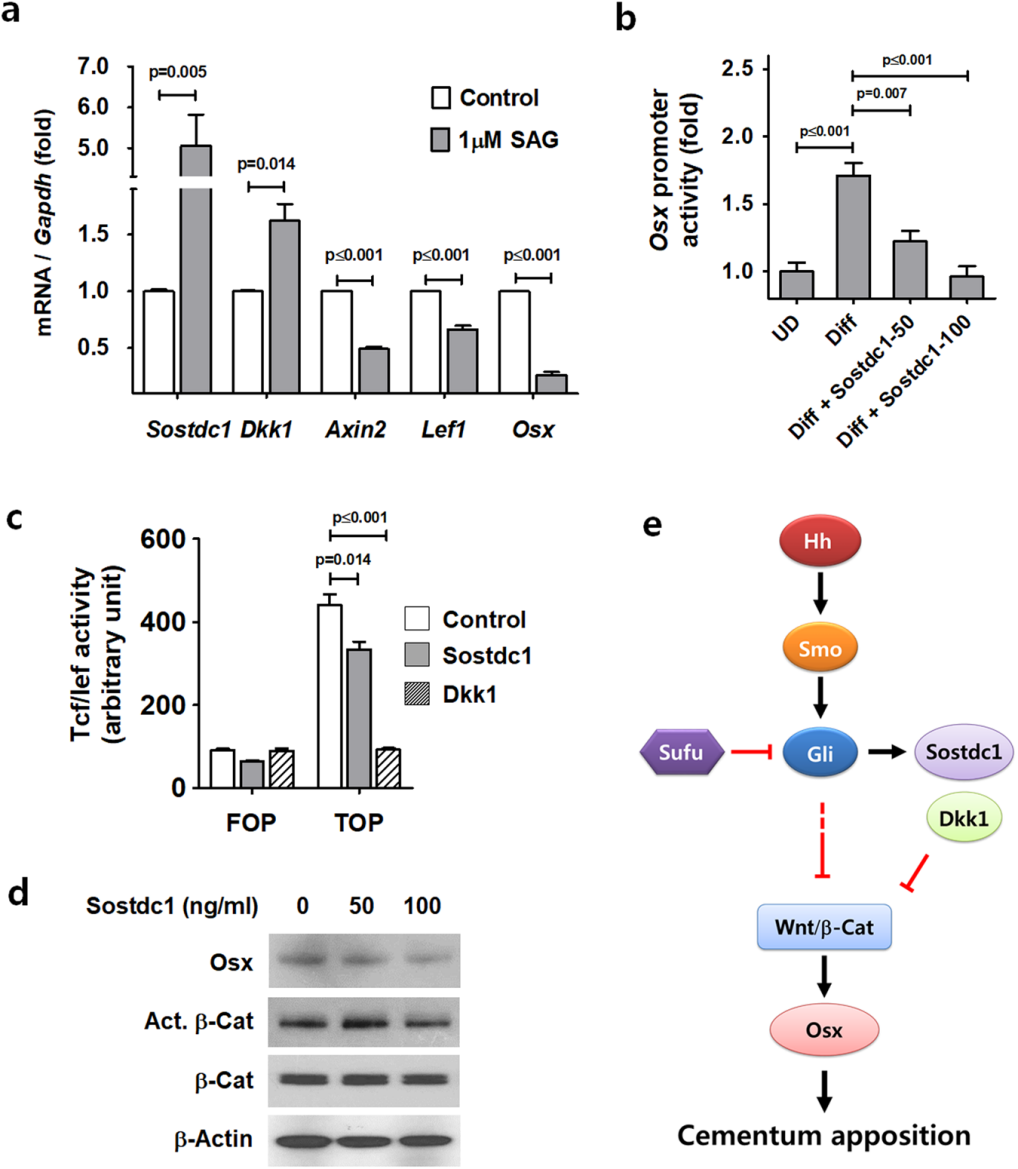
Sostdc1 activated by Hh signaling inhibits Osx expression and β-catenin activity in cementoblasts. (**a**) The mRNA transcript levels were analyzed by real-time qPCR. RNA was isolated from OCCM-30 cells treated with 1 μM SAG for 72 hours. **(b)** Luciferase activities driven by the *Osx* promoter were analyzed using OCCM-30 cellstreatedwith gradually increasing concentrations of recombinant Sostdc1 (50 and 100 ng/ml) for 48 hours in OM. UD, undifferentiated control. **(c)** Tcf/Lef binding activities of β-catenin were analyzed using OCCM-30 cells treated with OM and recombinant Sostdc1 (50 ng/ml) and Dkk1 (100 ng/ml) for 24 hours. **(d)** The protein levels of OCCM-30 cells treated with OM and indicated concentrations of SAG for 48 hours were analyzed by Western blotting. Samples shown in Western blotting are from the same experiment, and the gels/blots were processed under the same experimental conditions. β-Actin was used as a loading control. Cropped images aredisplayed here; the original full-size blots are presented in Supplementary Fig. S7. Act. β-catenin, Active β-catenin. Significance was assigned for *p*-values as indicated. **(e)** Schematic working model proposing the major hypothesis. We demonstrate that Hh-Smo signaling inhibits cementum apposition by repressing Wnt/β-catenin signaling and Osx expression and that possible downstream mediators of this process are Sostdc1 and Dkk1.

We found that the transcripts of *Sostdc1* and *Dkk1*, known targets of Hh signaling in tooth development^19,20^, were significantly increased with the down-regulation of *Axin2*, *Lef1*, and *Osx* in OCCM-30 cells treated with SAG (Fig. 5a). In accordance with the suppression of cellular cementum formation, the immunoreactivity of Sostdc1 around the apex of the tooth root was also increased(Supplementary Fig. S4a,b). To evaluate the suppressive effect of Sostdc1 on cementum formation *in vitro*, we analyzed *Osx* promoter activity after treatment of recombinant Sostdc1 to OCCM-30 cells with differentiation. *Osx* promoter activities were significantly decreased by recombinant Sostdc1 (Fig. 5b) and Dkk1 (Supplementary Fig. S5) treatment in a concentration-dependent manner, while they significantly increased by OM treatment for differentiation compared to the undifferentiated control (UD). Tcf/Lef binding activities of β-catenin were also decreased by the treatment of recombinant Sostdc1 and Dkk1 to OCCM-30 cells (Fig. 5c). In addition, the protein levels of Osx and non-phosphorylated β-catenin (Active β-catenin) were reduced by the treatment of recombinant Sostdc1 while the protein levels of total β-catenin and β-actin were not altered (Fig. 5d). Our data suggest that elevated Sostdc1 and Dkk1 driven by Hh-Smo signaling activation, at least in part, underlies the failure of the Wnt/β-catenin/Osx signaling pathway, leading to cementum apposition defects in mutant mice.

## Discussion

In this study, we investigated the role of Hh signaling during cementum development. We analyzed two kinds of cementoblast-specific Hh-Smo gain-of-function mouse models using Cre recombination system, an endogenous activation of Smo (*SmoM2*^*OC*^) and an inactivation of an antagonistic molecule, Sufu (*Sufu*^*OC*^). Thus, we provide genetic evidence that Hh-Smo signaling activation in cementoblasts results in a common defective formation of cellular cementum following down-regulation of matrix formation and mineralization ability. We also show that Hh signaling inhibits cementum apposition by repressing Wnt/β-catenin signaling and Osx expression and that possible downstream mediators of this process are Sostdc1 and Dkk1 (Fig. 5e).

The Gli family transcription factors are the major mediator of Hh signaling upon activation. Many of the studies have focused on how the Hh signal transduced by the receptor Smo induces the magnitude and quality of the resultant Hh-dependent target gene expression through the Gli transcription factors^21^. Sufu is one of repressors of Hh signaling by modulation of Gli transcription factors in mammals^13^. Ablation of *Sufu* in neural crest-derived mesenchymal cells leads to a defect in calvarial bone formation^16^. The transcript and protein levels of *Runx2* and *Osx*, the osteogenic regulators, were suppressed in calvarial mesenchyme of the *Wnt1-Cre*-mediated *Sufu* mutant mice. In addition, sustained Hh activity in osteoblasts inhibits postnatal bone development by suppressing the gene expression of regulatory factors such as Runx2, Osx, type I collagen, and OC in mice^17^. Unlike our understanding of bone formation with Hh signaling, the molecular mechanisms by which cementum formation can be regulated by Hh signaling in cementogenesis remain unclear. In this study, we demonstrated that Hh signaling negatively regulates cementum apposition by repressing Wnt/β-catenin signaling and Osx expression during tooth development. However, as analyzed with *Smo*^*OC*^ mutant mice, genetic *Smo* inactivation is not enough to promote cementum apposition *in vivo*. Interestingly, two Hh-Smo gain-of-function mouse models, *Sufu*^*OC*^ and *SmoM2*^*OC*^, showed clear distinction in reduction rate of cementum apposition. SmoM2 contains a point mutation, W539L, which renders it constitutively active leading to ligand-independent signaling^22^. As a common inhibitor of Hh and Wnt/β-catenin signals^15^, Sufu may be required for the integration of the two pathways during cementum development. *Sufu*^*OC*^ mutant exhibited weaker phenotypes in cellular cementum apposition than *SmoM2*^*OC*^ mutnat mice. Therefore, the results strongly suggest a crosstalk between Hh and Wnt/β-catenin signaling by Sufu in cellular cementum development.

Apical cellular cementumis generally developed after birth and also governed by the regulation of postnatally activated factors. Osx is one of key regulators in tooth root formation during development^4^. Previously, the spatiotemporal expression pattern of Osx have been intensely related with the formation of cellular cementum^5^. Furthermore, genetic modulation of Osx *in vivo* is positively related with the volume of cellular cementum^5^. In addition, we demonstrated that forced endogenous activation of β-catenin in cementoblasts robustly leads to the consequences of ectopic formation of cellular cementum^7^. Interestingly, Wnt/β-catenin activity and Osx expression in cementoblasts and cementocytes correlate with cellular cementum formation around root apex^5,23^. In this study, we analyzed Wnt/β-catenin/Osx signaling as an underlying mechanism governed by Hh signaling during cementogenesis. Finally, we demonstrate the complete restoration of apical cementum deformities shown in single Hh-Smo activation mutants by the compound mutation with endogenous activation of β-catenin. These results confirmed that Wnt/β-catenin signaling is adominant postnatal driving factor in addition to Osx for cellular cementum development.

It has been reported that Sostdc1 and Dkk1 are targets of Hh signaling in tooth, and *Sostdc1* knockout mice have elevated Wnt signaling in tooth development^20^. We identified and characterized Sostdc1 and Dkk1 as inhibitors of Osx expression in cementogenesis. Sostdc1 is a well-known inhibitor of the Wnt and BMP pathways^19,20,24^. Dkk1 is also known to be a negative regulator of Wnt signaling^25^. Our study suggests that Sostdc1 and Dkk1 contribute to cementum apposition defects in mutant mice as mediators to inhibit Wnt/β-catenin/Osx signaling axis.

Taken together, our results indicate a negative feedback loop between Hh-Smo and Wnt/β-catenin/Osx signaling during cementum formation and that a minimized level of Hh-Smo signaling is required for proper cellular cementum apposition. We provide new information about the molecular processes affecting cementum formation to improve our understanding and motivate the development of therapeutic approaches for patients.

## Materials and Methods

### Mice

All procedures were performed in accordance with the National Institutes of Health Guidelines on the Use of Laboratory Animals. All experimental procedures were approved by the Animal Welfare Committee of Chonbuk National University. All the mice were housed in a temperature-controlled environment with 12 h light/dark cycles. We generated a conditionally regulated mouse by crossing an activated allele mice of Smoothened, *SmoM2*^22^ and *Sufu*-floxed (*Sufu*^*fl/fl*^)^26^ mice with *Osteocalcin* (*OC*)-Cre transgenic mice^27^. *Catnb*^*lox(ex3)/+*^(*Catnb*^*lox/+*^) has been previously described^28^. To generate *OC-Cre:Catnb*^*lox/+*^:*Sufu*^*fl/fl*^(*Catnb:Sufu*^*OC*^) mice, *OC-Cre;Sufu*^*fl/+*^ mice were crossed with *Catnb*^*lox/+*^:*Sufu*^*fl/fl*^ mice. To generate *OC-Cre:Catnb*^*lox/+*^:*SmoM2*(*Catnb:SmoM2*^*OC*^) mice, *OC-Cre* mice were crossed with *Catnb*^*lox/+*^*:SmoM2* mice. We also analyzed conditionally inactivated allele mice of Smoothened, *Smo*^*OC*^ under the same Cre regulation^29^. The offspring were genotyped by polymerase chain reaction (PCR) analysis using previously described primers. *Sufu*^*fl/fl*^ or *SmoM2* mice were used as controls. At least three independent littermates were used for each experimental group (n ≥ 5/genotype, including males and females, age indicated in the figure).

### Micro-CT analysis and double-fluorochrome labeling

For micro-CT (μCT) analysis, the mandibles were dissected at P28, bisected at the symphysis, and fixed in 4% PFA (Sigma Aldrich, St. Louis, MO, USA). The mandibles were scanned using a desktop scanner (1076Skyscan Micro-CT, Skyscan, Kontich, Belgium) and analyzed using the CTAn software (Skyscan). For double-fluorochrome labelling, calcein (20 mg/kg of body weight intraperitoneally; Sigma Aldrich) was injected twice at P28 and P56. The mice were sacrificed at P58. Fifty-micrometer cross-sections were cut perpendicularly, passing through the midsection of the mandibular first molar, and viewed under a model LSM510 confocal laser scanning microscope (Carl Zeiss, Ostalbkreis, Germany). Cellular cementum thickness (μm) was measured as the shortest vertical distances beginning from the fluorochrome-labeled proximal line to another distal line using the analySIS Pro imaging system (Soft Imaging System). Eight start points of the proximal line for measurement were randomly selected within the apical part of the root. For thickness evaluation, the average cementum mineral apposition rate (μm/day) was acquired as the distances divided by the number of days between injections. The experiments were performed three times with representative slides from each group, and the differences of the values were evaluated statistically (*p* < 0.001).

### Immunohistochemistry (IHC) and histomorphometry

The sections were treated with 3% hydrogen peroxide and incubated with rabbit polyclonal antibodies against bone sialoprotein (Bsp; Abcam, Cambridge, MA, USA), β-catenin (Thermo Scientific, Fremont, CA, USA), Osx (Santa Cruz Biotechnology, Dallas, TX, USA), Dmp1 (TaKaRa Bio, Shiga, Japan), Axin2 (Abcam), Sufu (Proteintech, Rosemont, IL, USA), Ptc1 (Abcam), and Sostdc1 (Abcam). The HistostainPlus Rabbit Primary (DAB) kit (Zymed Laboratories, San Francisco, CA, USA) was used following the manufacturers’ instructions. The average cellular cementum area was calculated usingthree measurements of five representative individual slides in each group at the indicated age using the analySIS Pro imaging system (Soft Imaging System, Münster, Germany).

### Cell culture and treatment

OCCM-30, a mouse cementoblast cell line, was provided by Dr. Martha J. Somerman (National Institutes of Health, Bethesda, MD, USA) and cultured as described previously^30^. SAG (N-Methyl-Nʹ-(3-pyridinylbenzyl)-Nʹ-(3-chlorobenzo[b]thiophene-2-carbonyl)-1,4-diaminocyclohexane; Calbiochem, San Diego, CA, USA), a cell-permeable Smo agonist, was used to treat cells under the concentration of 1μM for up to 72 hours, in which cell toxicity was not detected. To induce cell differentiation and mineralization, 95% confluent cells were cultured in osteogenic medium (OM), which consisted of the medium supplemented with 2% fetal bovine serum, 50 μg/ml ascorbic acid (Sigma Aldrich), and 10 mM β-glycerophosphate (Sigma Aldrich), for up to 4 days. Recombinant mouse Sclerostin domain-containing protein 1 (Sostdc1; R&D, Minneapolis, MN, USA) and Dickkopf related protein 1 (Dkk1; Biolegend, San Diego, CA, USA) were used to treat cells at the indicated concentrations for 24 or 48 hours in osteogenic medium.

### Statistical analysis and Supplemental information

Data are presented as mean±standard error of the mean (SEM) of three or more separate experiments. Normal data with equal variance were analyzed using Student’s t-test and *p* < 0.05 was considered statistically significant. Detailed descriptions for other experimental materials and methods are provided in Supplementary Methods.

## Supplementary information

Supplementary information.
