# Supplementary material for: Suppression of Hedgehog signaling is required for cementum apposition
Source: Sci Rep. 2020 Apr 29;10:7285. doi: 10.1038/s41598-020-64188-w (PMC7190817; doi:10.1038/s41598-020-64188-w)

Supplementary Information

Suppression of Hedgehog signaling is required for cementum apposition

Hwajung Choi[1,#] , Yudong Liu[1,2,#], Liu Yang[1], Eui-Sic Cho[1,*]

[1]Cluster for Craniofacial Development and Regeneration Research, Institute of Oral Biosciences, Chonbuk National University School of Dentistry, Jeonju 54896, South Korea

[2]Department of Histology and Embryology, Bengbu Medical College, Bengbu, Anhui, P.R. China

Supplementary Table S1. Primer sequences for real-time qPCR

Gene	Sense	Antisense
Gli1	GTCGGAAGTCCTATTCACGC	CAGTCTGCTCTCTTCCCTGC
Gli2	AAGCACCAGAACCGCACTCACTC	CTTGAGCAGTGGAGCACGGACAT
Ptc1	AACAAAAATTCAACCAAACCTC	TGTCTTCATTCCAGTTGATGTG
Sufu	GGAGCCCTCATCCCTCTCTGCCTAA	TACGGGTGTTCCTCAGTGGCAAAGG
Osx	TCTCCATCTGCCTGACTCCT	AGCGTATGGCTTCTTTGTGC
Bsp	AAAGTGAAGGAAAGCGACGA	GTTCCTTCTGCACCTGCTTC
Oc	ACCCTGGCTGCGCTCTGTCTCT	GATGCGTTTGTAGGCGGTCTTCA
Axin2	AAGAAGGAGACCGGTCACAG	GGTCCTGGGTAAATGGGTGA
Lef1	TTCAGGTACAGGTCCCAGAATG	AGTCGGCGCTTGCAGTAGA
Col1a1	CCGGAAGAATACGTATCACC	ACCAGGAGGACCAGGAAGTC
Col1a2	CAGCGAAGAACTCATACAGCC	TTGGAGCAGCCATCGACTA
Opn	CCCGGTGAAAGTGACTGATTC	ATGGCTTTCATTGGAATTGC
Sostdc1	CATGCTTCCTCCTGCCATTC	TACTGAAATGCCTGCCTCCA
Dkk1	TCAATTCCAACGCGATCAAGA	GGCTGGTAGTTGTCAAGAGTCTGG
Gapdh	TGCCCAGAACATCATCCCT	GGTCCTCAGTGTAGCCCAAG

Supplementary Methods

Tissue preparation and histology

For histologic analysis, mice were sacrificed and their mandibles were carefully dissected. The dissected tissues were fixed in 4% paraformaldehyde (PFA; Sigma Aldrich, St Louis, MO, USA) and decalcified in 10% EDTA for 2 to 4 weeks at 4°C. The decalcified tissues were dehydrated

through a graded ethanol series, embedded in paraffin, and sectioned at 5-μm thickness. Slides were stained with hematoxylin and eosin (H-E).

RNA preparation and real-time qPCR

Total RNA was prepared using an RNeasy Mini kit (QIAGEN, Valencia, CA, USA) according to the manufacturer's instructions, and cDNA was synthesized from 3 μg of total RNA using Superscript II reverse transcriptase (Invitrogen). Real-time PCR was performed with SYBR Green PCR Master Mix (Applied Biosystems, Warrington, Cheshire, UK) following the manufacturer's protocols. Reaction conditions comprised 40 cycles of 15 seconds of denaturation at 95℃ and 1 min of amplification at 60℃. All reactions were run in triplicate; expression was normalized to that of the housekeeping gene *glyceraldehyde-3-phosphate dehydrogenase* (*Gapdh*). Relative levels of transcript expression were quantified using the $\Delta\Delta Ct$ method. The calculation was performed using the *Ct* value of *Gapdh* to normalize the *Ct* value of the target gene in each sample and obtain the ΔCt value, which then was used to compare different samples. Relative mRNA expression was compared in a histogram. Specific primer sets used in the analysis are listed in Supplementary Table S1.

Western blot analysis

Whole cell proteins (30 μg) were dissolved in sample buffer and resolved by electrophoresis with a current of 25 mA for 2 hours. Proteins were transferred from the SDS-PAGE gels onto PVDF membranes (Schleicher & Schuell, Dassel, Germany). Nonspecific binding sites on membranes were blocked for 1 h with 5% nonfat dry milk in PBS (blocking buffer) and incubated overnight with IgG antibodies against Gli1 (1:500, LifeSpan BioSciences, Inc., Seattle, WA, USA), non-phosphorylated (active) β-catenin (1:1000, Cell Signaling), β-catenin

(1:2000, Thermo Scientific), Osx (1:500, Santa Cruz Biotechnology), and β-actin (1:2000, Santa Cruz Biotechnology) diluted in blocking buffer at 4 °C. After washing, the membranes were incubated with horseradish peroxidase-conjugated anti-rabbit or anti-mouse IgG antibodies (Santa Cruz Biotechnology) in blocking buffer at room temperature for 1 hour. Immunoreactive bands were detected using an enhanced chemiluminescence system (Amersham Biosciences, Buckinghamshire, UK). Protein expression levels were analyzed with the ImageQuanT TL 1D gel analysis program (Amersham Biosciences).

Transfection and luciferase activity

The plasmid driving the expression of mouse β-catenin S33Y was a gift from Shinya Yamanaka (Addgene plasmids #13371). Flag-tagged mouse *Osx* construct (accession no. NM_130458) in the pCMV6 backbone was purchased from OriGene Technologies (Rockville, MD, USA). The luciferase *Osx* promoter plasmid -1269/+91 was a gift from Dr. Mark Nanes (Emory University, Atlanta, GA, USA). TOPflash/FOPflash reporter (Addgene ##12456 and #12457, respectively) constructs were used for Tcf/Lef binding activity. DNA constructs were transfected by using Lipofectamine™ LTX and PLUS reagent (Invitrogen) according to the manufacturer's instructions. Luciferase activity was determined using the Dual-Luciferase reporter assay system (Promega, Madison, WI, USA), according to the manufacturer's instructions. Light intensity was measured with a luminometer, and the luciferase activity was divided by that of the control reporter to normalize for transfection efficiency.

Mineralization induction and alizarin red staining

To induce cell differentiation and mineralization, 95% confluent cells were cultured in osteogenic medium (OM). Mineral nodule formation was then observed by staining the cells

with 40 mM alizarin red S (pH 4.2) after fixation with 4% PFA for 10 minutes. The amount of alizarin red S that bound to the minerals was quantified by destaining the samples in 10 mM sodium phosphate containing 10% cetylpyridinium chloride (pH 7.0) for 15 minutes at room temperature. The amount of alizarin red S in the destaining solution was measured at OD 562 nm.

Alkaline phosphatase (ALP) activity, ALP staining and Trichrome staining

Cells were cultured with or without SAG in OM for 48 hours. Alkaline phosphatase activity was quantitated using an assay based on the hydrolysis of p-nitrophenylphosphate (p-NPP) to p-nitrophenol (p-NP). Briefly, cell layers were washed twice with ice-cold PBS and lysed in 50 mM Tris–HCl buffer (pH 7.0) containing 1% (v/v) Triton X-100 (Sigma Aldrich) and 1 mM PMSF (Sigma Aldrich). Whole cell lysates were assayed by adding 1 mg/ml of pNPP substrate in 0.1 M glycine buffer (pH 10.4) containing 1 mM $ZnCl_2$ (Sigma Aldrich) and 1 mM $MgCl_2$ (Sigma Aldrich) to each tube for 15 min at 37 ℃. Reactions were stopped by adding NaOH (final concentration 0.6 N), and the absorbance of each lysate was measured spectrophotometrically at 405 nm. Enzyme activity was normalized to total protein content and expressed using the fold change. For ALP staining, cells were stained with a Leukocyte Alkaline Phosphatase kit (Sigma Aldrich) according to the manufacturer's protocol. Trichrome Stain Kit (Sigma Aldrich) was used for connective tissue stain according to the manufacturer's protocol.

TRAP staining and ImageJ

To detect osteoclasts, TRAP staining was performed with a TRAP staining kit (Sigma-Aldrich). ImageJ (http://imagej.nih.gov/ij, NIH, USA) was used to determine the positive area of TRAP

staining and IHC results. The TRAP-positive (TRAP+) area were analyzed using the 'Color Threshold' function of image adjustment. The signals in the cementum and alveolar bone of molar apical regions were separately measured and calculated after setting scale. The numbers of TRAP+ cells in the cementum and alveolar bone were separately counted by using microscopy. Positive signals of IHC results were measured using the 'Color Threshold' function of image adjustment after 'Split Channels' for color.

Legends for Supplementary Figures

Supplementary Figure S1. Molecular changes of Sufu and Ptc1 following conditional *Sufu* inactivation. (a and b) Molecular changes of Sufu **(a)** and Ptc1 **(b)** in the apical cementum (indicated by dotted lines) were detected by IHC staining with the distal root of the mandibular first molar from $Sufu^{OC}$ mutant and the control mice at P28. Sufu expression in the developing cementum of control mice, especially at cementocytes and cementoblasts, was detected while barely detected in $Sufu^{OC}$ mice. An increased Ptc1, a downstream target of Hh signaling, immunoreactivity confirmed the Hh gain-of-function in the dental tissue of $Sufu^{OC}$ mice. The lower images are higher-magnification views of apical cementum in the boxed area of top images. The arrows indicates reduced expression of Sufu (blue) and increased expression of Ptc1 (red), respectively. C, cementum; D, dentin; PDL, periodontal ligament. Scale bars: 50 μm. **(c and d)** Positive signals of above IHC results for Sufu **(c)** and Ptc1 **(d)** and the comparable counterstain (Hematoxylin) were measured.

Supplementary Figure S2. Morphological changes of the tooth with Hh signaling activation and inactivation. (a) Morphological changes in dentin thickness of $Sufu^{OC}$,

SmoM2^{OC} mutant and the control mice were compared with the mandibular first molar by H-E staining at P14. The lower images are higher-magnification views of crown dentin in the boxed area of top images. D, dentin; Od, odontoblasts. Scale bars: 400 μm (top) and 100 μm (below). **(b)** Morphological changes in the apical cellular cementum (indicated by dotted lines) of *Sufu*^{OC}, *SmoM2*^{OC} mutant and the control mice were compared with the distal root of the mandibular first molar by H-E staining at P28 and P56 of age. C, cementum; D, dentin; PDL, periodontal ligament. Scale bars: 50 μm. **(c)** Morphological changes of *SmoM2*^{OC} and *Smo*^{OC} mutant and the control mice were compared with the mandibular first molar by Trichrome staining at P28. Scale bars: 400 μm.

Supplementary Figure S3. Postnatal resorption process was not involved in the morphological changes of the apical cementum in Hh-Smo signaling activation mutant mice. (a) TRAP staining was performed to analyze osteoclastogenesis with the tissue sections of the distal root of the first molar at P28. Blue arrows indicate the negative staining of TRAP around reduced cellular cementum (indicated by dotted lines). Red arrows indicate the positive staining of TRAP at the marginal area of alveolar bone neighboring the apical tooth root. D, dentin; C, cementum; PDL, periodontal ligament; AB, alveolar bone. Scale bar; 100 μm. **(b and c)** TRAP positive area **(b)** and cell number **(c)** were analyzed with the TRAP-stained tissue section.

Supplementary Figure S4. Molecular changes of Sostdc1 in the apical cementum with Hh signaling activation. (a) Molecular changes of Sostdc1 in the apical cementum (indicated by dotted lines) were detected by immunohistochemical staining with the distal root of the mandibular first molar at P28. Scale bars: 100 μm. **(b)** Positive signals of above IHC results

for Sostdc1 and the comparable counterstain (Hematoxylin) were measured.

Supplementary Figure S5. Dkk1 activated by Hh signaling regulates Osx expression in cementoblasts. Luciferase activities driven by the *Osx* promoter were analyzed using OCCM-30 cells treated with OM and gradually increasing concentrations of recombinant Dkk1 for 24 and 48 hours, respectively. Significance was assigned for *p*-values as indicated. UD, undifferentiated control.

Supplementary Figure S6. Original full-size blots of Figure 2b and 3d. (a) The following antibodies were used: Gli1 (1:500; 180157, LifeSpan BioSciences, Inc.), and β-Actin (1:2000, sc-1616R, Santa Cruz Biotechnology). **(b)** The following antibodies were used: Gli1 (1:500; 180157, LifeSpan BioSciences, Inc.), Osx (1:500; sc-22536, Santa Cruz Biotechnology), and β-Actin (1:2000, sc-1616R, Santa Cruz Biotechnology).

Supplementary Figure S7. Original full-size blots of Figure 5d. The following antibodies were used: Osx (1:500; sc-22536, Santa Cruz Biotechnology), active β-catenin (1:1000; 8814, Cell Signaling), β-catenin (1:2000; RB-9031, Thermo Scientific), and β-Actin (1:2000, sc-1616R, Santa Cruz Biotechnology).

Supplementary Figure S1

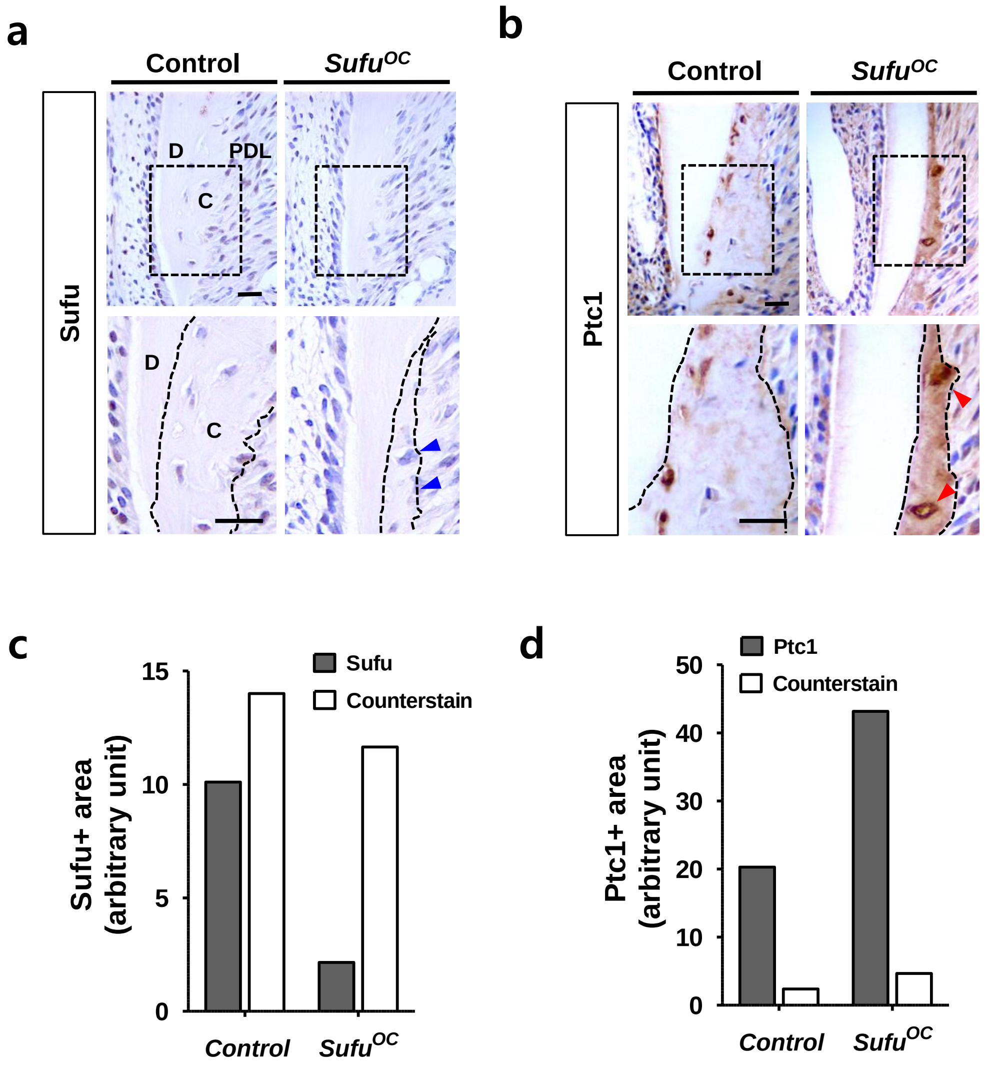

Supplementary Figure S2

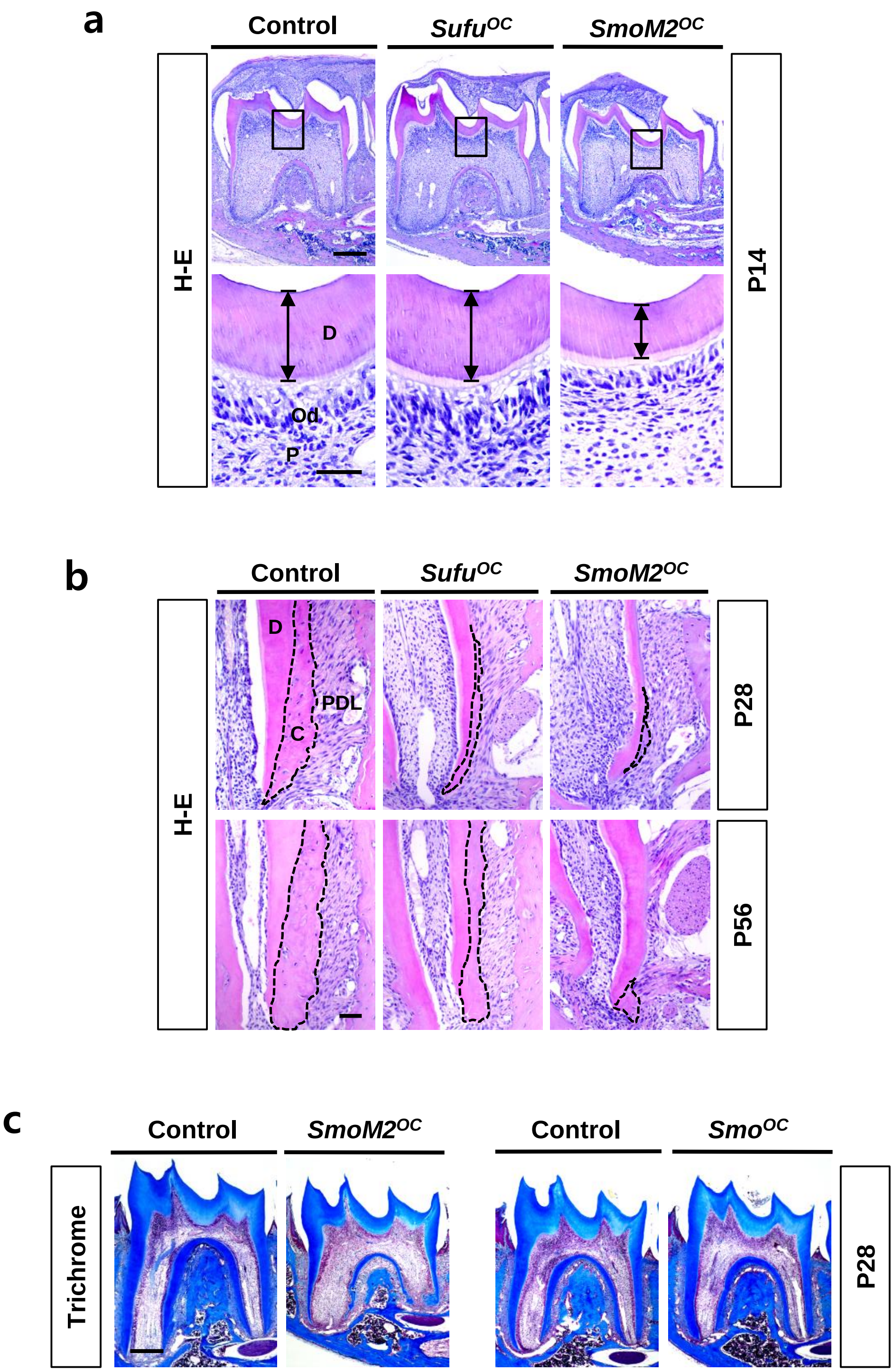

Supplementary Figure S3

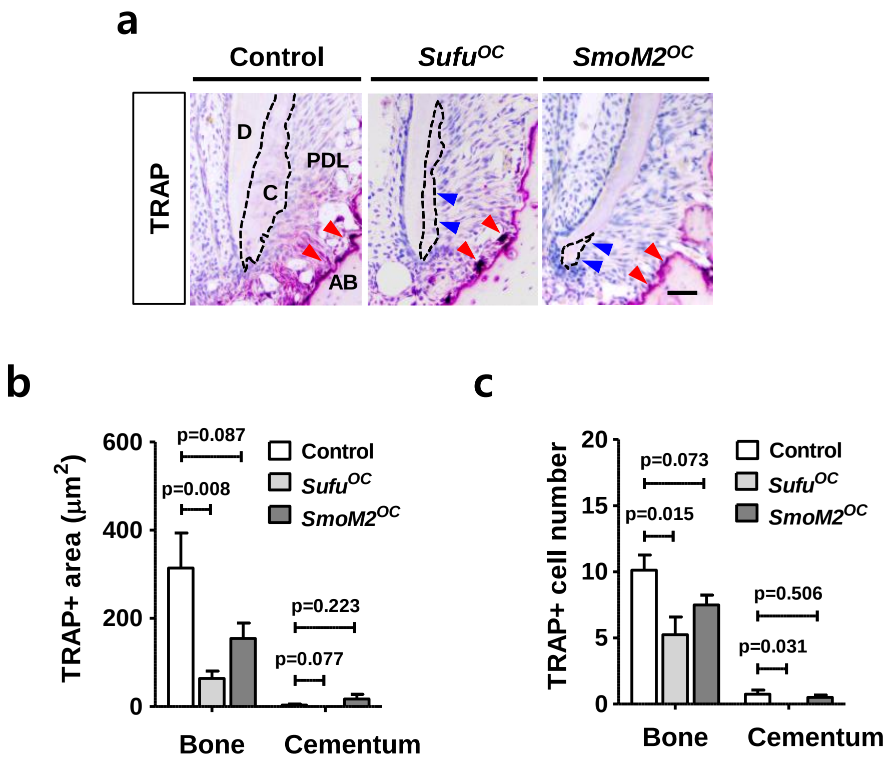

Supplementary Figure S4

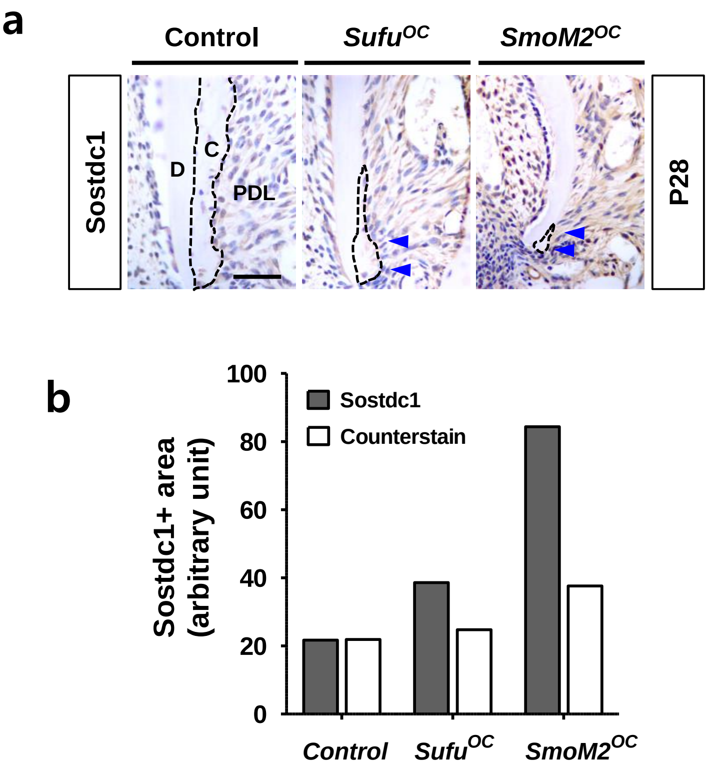

Supplementary Figure S5

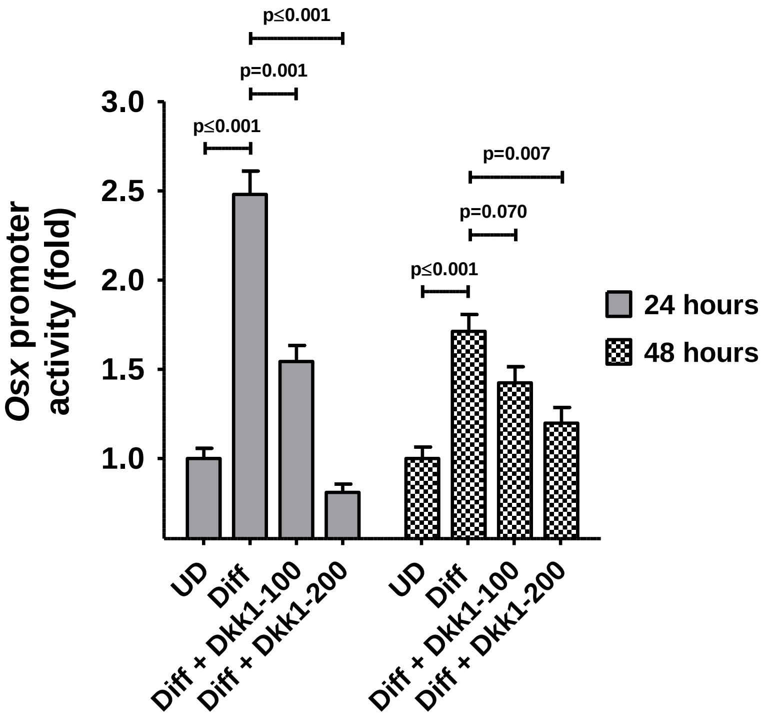

Supplementary Figure S6

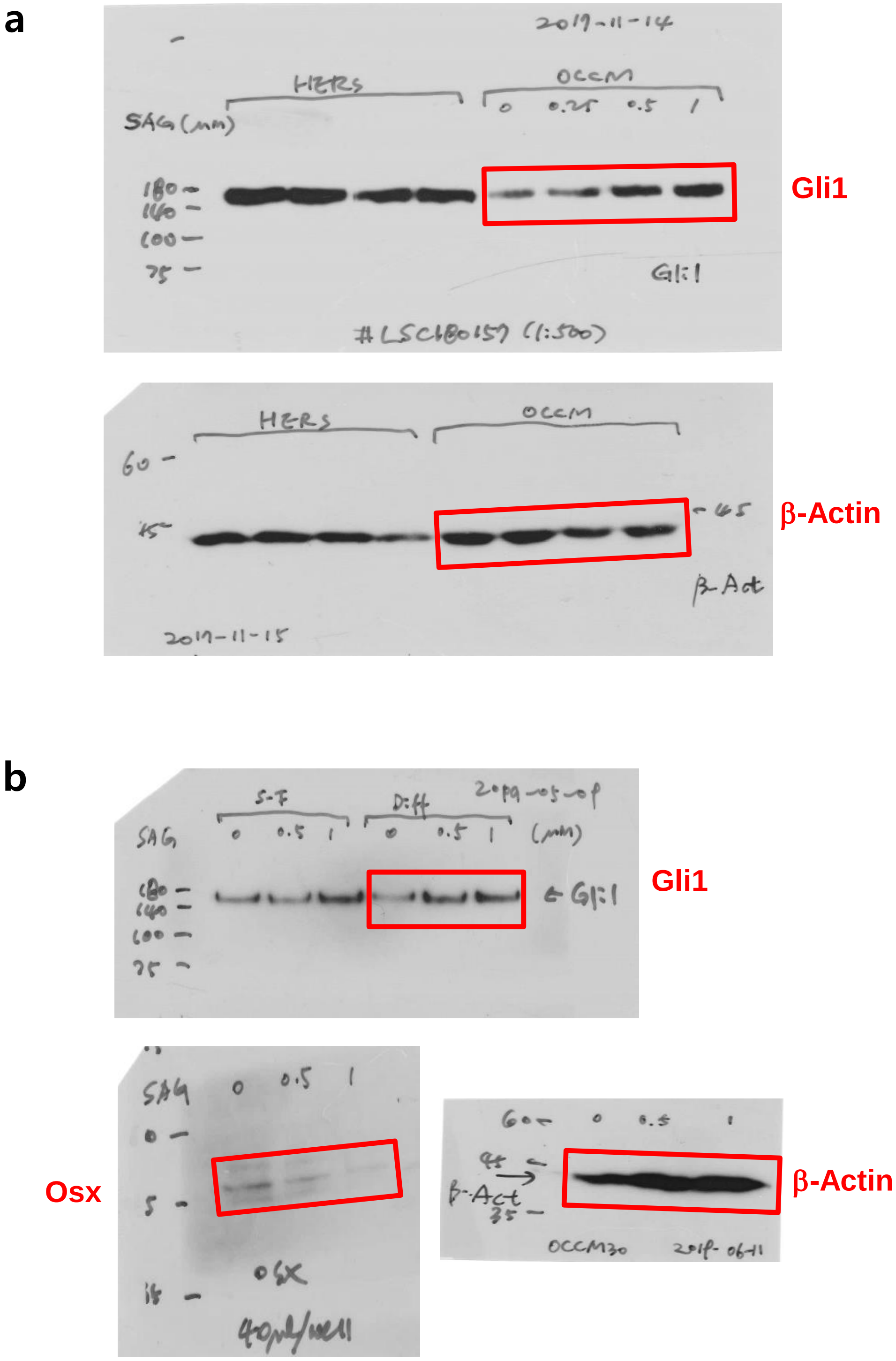

Supplementary Figure S7

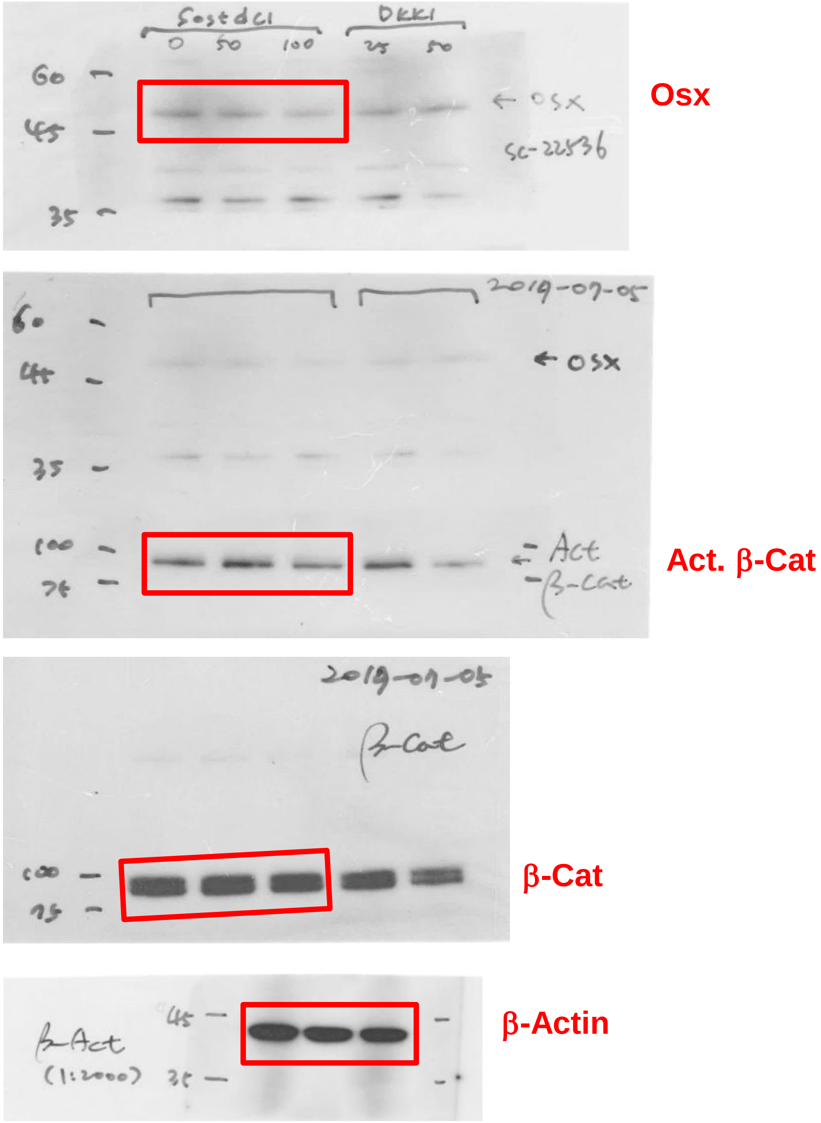

Supplement: Supplementary file 1 — Supplementary information. [file 41598_2020_64188_MOESM1_ESM.pdf]
